# Identification of CKS2 and RRM2 as potential markers of vitiligo using bioinformatics analysis

**DOI:** 10.1097/MD.0000000000031908

**Published:** 2022-11-18

**Authors:** Yu Miao, Dongqiang Su, Qian Fu, Taoyu Chen, Yanqi Ji, Feng Zhang

**Affiliations:** a Department of Dermatology, First Affiliated Hospital of Harbin Medical University, Harbin, Heilongjiang Province, China; b Department of Dermatology, Sixth Affiliated Hospital of Harbin Medical University, Harbin, Heilongjiang Province, China.

**Keywords:** bioinformatics, immune cell infiltration, vitiligo

## Abstract

Previous studies have attempted to elucidate the molecular mechanism of vitiligo; however, its pathogenesis remains unclear. This study aimed to explore biomarkers related to vitiligo through bioinformatic analysis. The microarray datasets GSE53146 and GSE65127 were downloaded from the Gene Expression Omnibus database. Firstly, differentially expressed genes (DEGs) in GSE53146 were screened, and then an enrichment analysis was performed. Secondly, the protein-protein interaction (PPI) network of DEGs was constructed using the STRING database, and the key genes were screened using the MCODE plugin in Cytoscape and verified using GSE65127. Finally, quantiseq was used to evaluate immune cell infiltration in vitiligo, then to observe the correlation between biomarkers and immune cells. In total, 544 DEGs were identified, including 342 upregulated and 202 downregulated genes. Gene Ontology (GO) enrichment showed that DEGs were related to inflammatory and immune responses, and Kyoto Encyclopedia of Genes and Genomes enrichment showed that DEGs were involved in many autoimmune diseases. In the PPI network, 7 key genes, CENPN, CKS2, PLK4, RRM2, TPX2, CCNA2, and CDC45 were identified by MCODE cluster and verified using the GSE65127 dataset. With an area under the curve (AUC) > 0.8 as the standard, 2 genes were screened, namely CKS2 and RRM2. Further immune infiltration analysis showed that M2 macrophages were involved in the pathogenesis of vitiligo, whereas CKS2 and RRM2 were both related to M2 macrophages. This study shows that CKS2 and RRM2 have potential as biomarkers of vitiligo and provides a theoretical basis for a better understanding of the pathogenesis of vitiligo.

## 1. Introduction

Vitiligo is an autoimmune disease that affects the skin and is characterized by white patches caused by the loss of epidermal melanin.^[1]^ Vitiligo is less likely to be life-threatening, so it seems to belong to a low-priority medical area; however, because the rash easily occurs in significant parts, it usually causes negative effects such as shame, fear, anxiety, and discrimination.^[1]^ Studies have shown that the decline in quality of life caused by reduced self-esteem and social isolation in patients with vitiligo can be comparable to that of psoriasis,^[2]^ so we should be paid to this disease. The current treatment can reverse vitiligo by stimulating the regeneration of the melanocyte stem cell reservoir in the hair follicle and suppressing the immune response to repigment the skin lesions,^[3]^ but the risk of recurrence in the first year is approximately 40%.^[4]^ In addition, different patients and rashes in different parts have different sensitivities to treatment, and their efficacy is uneven.^[5]^ Therefore, it is very important to deeply understand the complex pathogenesis of vitiligo, develop new treatment methods, and meet the treatment needs of patients.

Bioinformatics has developed rapidly since the 21st century and has become a promising analytical method. The differentially expressed genes (DEGs) between the diseased population and healthy controls were screened, and the potential pathogenic pathways were verified, providing a theoretical basis for an in-depth understanding of the molecular mechanism of the disease, which can be further used for disease screening, prognosis, targeted therapy, and drug development.^[6]^ High-throughput sequencing and DNA microarrays can provide genome-wide expression data; therefore, they are widely used in the medical field. These data are stored in public databases, such as Gene Expression Omnibus (GEO), The Cancer Genome Atlas (TCGA),^[7]^ and Online Mendelian inheritance in humans (OMIM).^[8]^ In this study, we downloaded microarray data on vitiligo from the GEO database and screened key genes to explore the genes related to the pathogenesis of vitiligo through bioinformatics analysis and provide direction for disease prediction and accurate treatment.

## 2. Materials & methods

### 2.1. Select dataset

All data were downloaded from the GEO database (https://www.ncbi.nlm.nih.gov/geo). Taking “vitiligo” AND “Homo sapiens” as the inclusion criteria, taking the tissue source as “skin biopsy,” and the sample containing “healthy control and disease group” as the screening criteria, 2 datasets, GSE53146 and GSE65127, were obtained. The sequencing platforms were GPL14951 (Illumina HumanHT-12 WG-DASL V4.0 R2 expression beadchip) and GPL570 (Affymetrix Human Genome U133 Plus 2.0 Array). The sequencing type used was microarray. Basic information on the dataset is provided in Table 1.

**Table 1:** Characteristics of dataset.

Dataset	Source	Type	Number of samples	Sample name
GSE53146	Skin biopsy	Vitiligo	5	GSM1283009~GSM1283013
		Healthy control	5	GSM1283004~GSM1283008
GSE65127	Skin biopsy	Vitiligo	10	GSM1587709~GSM1587718
		Healthy control	10	GSM1587719~GSM1587728

### 2.2. Data processing

R (https://www.r-project.org/) is a free and open-source programming language that can be run and compiled for different operating systems. The R package can be downloaded for statistical calculations and data visualization. RStudio (https://www.rstudio.com), as an editor of the R language, has the advantages of a simple page and convenient application.

This study primarily used R (v4.0.4) and RStudio (v1.4.1106) to analyze the dataset. The GSE53146 dataset was preprocessed using the limma package of R and visualized using R’s boxplot function.

### 2.3. Principal component analysis

Principal component analysis (PCA) is a statistical method used to study the relationships between multiple variables. It is one of the most widely used data dimensional-reduction algorithms. The basic principle is to reduce the dimensions of the dataset while retaining the characteristics that contribute the most to the difference in the dataset.

In this study, PCA was used to observe the gene expression patterns of the 2 groups, the prcomp function of R was used for calculation, and the ggord package was used for visualization to evaluate the difference between the samples of the vitiligo and healthy control groups.

### 2.4. Screen for differential genes

In this study, the annotation file was obtained from the corresponding sequencing platform, and the dataset was probe-transformed using the dplyr and tidyr packages of R. The limma package of R was used for calculation based on the processed GSE53146 dataset, and DEGs were identified and screened. The screening threshold was *P* < .05 and |logFC| > 2. The results are presented in the form of a heatmap and volcano map that were drawn using the pheatmap package and ggplot2 package of R, respectively.

### 2.5. Enrichment analysis

DAVID (https://david.ncifcrf.gov) is an online database that provides biological function annotation information for a large number of genes and proteins, helping researchers extract the most significant biological information from thousands of associated annotations. At present, it is mostly used for DEG function and pathway enrichment analyses.

Gene Ontology (GO) can define and describe the functions of genes and proteins. It divides functions into 3 parts: biological process (BP), cellular component (CC), and molecular function (MF). The Kyoto Encyclopedia of Genes and Genomes (KEGG) is a database that stores health information, such as the interaction between genomes and chemical substances, diseases, and drugs.

In this study, the DAVID database (v 6.8) was used for the GO and KEGG enrichment analyses of the screened DEGs. Statistical significance was set at *P *< .05. The obtained results were imported into R and visualized using the ggplot2 package.

### 2.6. Construct protein-protein interaction network and screen key genes

In this study, the STRING (https://cn.string-db.org, v 11.5) database was used to construct protein-protein interaction networks (PPI) of DEGs. The minimum interaction score was set to 0.7, the nodes that had no connection with other nodes were hidden, and Cytoscape (https://cytoscape.org, v 3.8.0) was used for visualization.

To identify genes that play a key role, namely hub genes, the MCODE plugin of Cytoscape was used to cluster and construct functional modules. The criteria for cluster analysis were mcode score > 2, degree cutoff = 2, node score cutoff = 0.2, k-core = 2, and max.depth = 100, and the genes involved in the central module were selected.

### 2.7. Dataset validation

The receiver operating characteristic (ROC) curve is often used for disease diagnosis, tumor recurrence, and treatment target prediction. By comparing the area under the curve (AUC), we can determine the best diagnostic value.

In this study, the hub genes were verified using the GSE65127 dataset. The pROC package of R was used for calculation and drawing, and genes with an AUC > 0.8 were selected.

### 2.8. Immune cell infiltration analysis

Immune cells are important for the innate and adaptive immune responses of organisms. They are diverse and play important roles in the human body. Therefore, in this study, we further examined whether there were differences in the distribution of immune cells in samples from vitiligo patients and healthy controls. We used the immunedeconv package of R and the quantiseq method to evaluate immune infiltration and drew a bar graph using the ggplot2 package. The differences in the expression of immune cells in the 2 groups of samples were compared, the rank sum test analysis was performed using R, and the ggplot2 package was used to draw histograms. Statistical significance was set at *P *< .05.

### 2.9. The connection between genes and immune cells

The distribution and function of these molecules are inseparable from gene regulation. Therefore, this study further explored whether the screened genes (genes with an AUC > 0.8) were related to immune cells. The correlation was calculated using the psych package in R, and the ggcorrplot package was used to plot the correlation.

## 3. Results

### 3.1. GSE53146 standardization and PCA analysis

**Figure 1.**
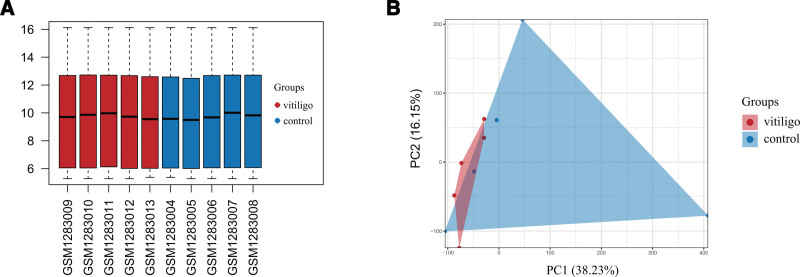
Data preprocessing and evaluation. (A) After standardization, the overall expression level of samples in the microarray. (B) PCA of GSE53146 samples. PCA = principal component analysis.

The GSE53146 dataset used in this study was downloaded from the GEO database, including sequencing information of skin lesions of 5 vitiligo patients and 5 healthy controls. After standardization using the limma package in R, the data distribution between the 2 groups of samples was relatively consistent, and there was no obvious deviation, as shown in Figure 1A. To further verify the dispersion of the different samples, PCA was performed. The results showed that the distance between the 5 groups of disease samples was close and the difference was small, and the distance between the 5 groups of control samples was far and the difference was large. There was a spatial separation between the vitiligo and control samples, as shown in Figure 1B.

### 3.2. Identification of DEGs

**Figure 2.**
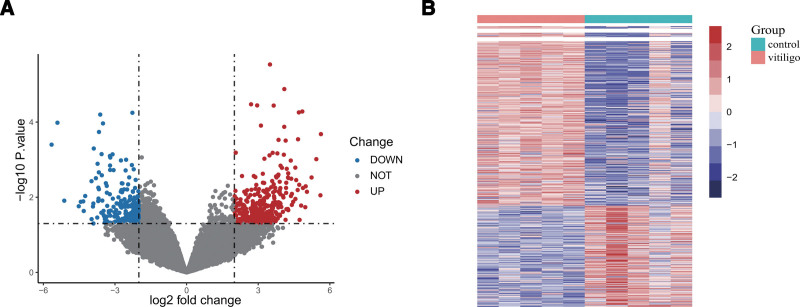
Identification of DEGs in GSE53146 dataset. (A) Volcano plot showing DEGs in vitiligo patients. Each dot represents a gene, in which blue, gray and red represent down-regulated, no difference and up-regulated genes respectively. (B) Heatmap showing the distribution of DEGs between different groups. DEGs = differentially expressed genes.

DEGs analysis of GSE53146 was performed using the limma package, and a total of 544 significant DEGs were detected, including 342 upregulated genes (UP) and 202 downregulated genes (DOWN), as shown in Figure 2A. The distribution of DEGs in the 2 groups is presented as a heat map (Fig. 2B).

### 3.3. Enrichment analysis of DEGs

**Figure 3.**
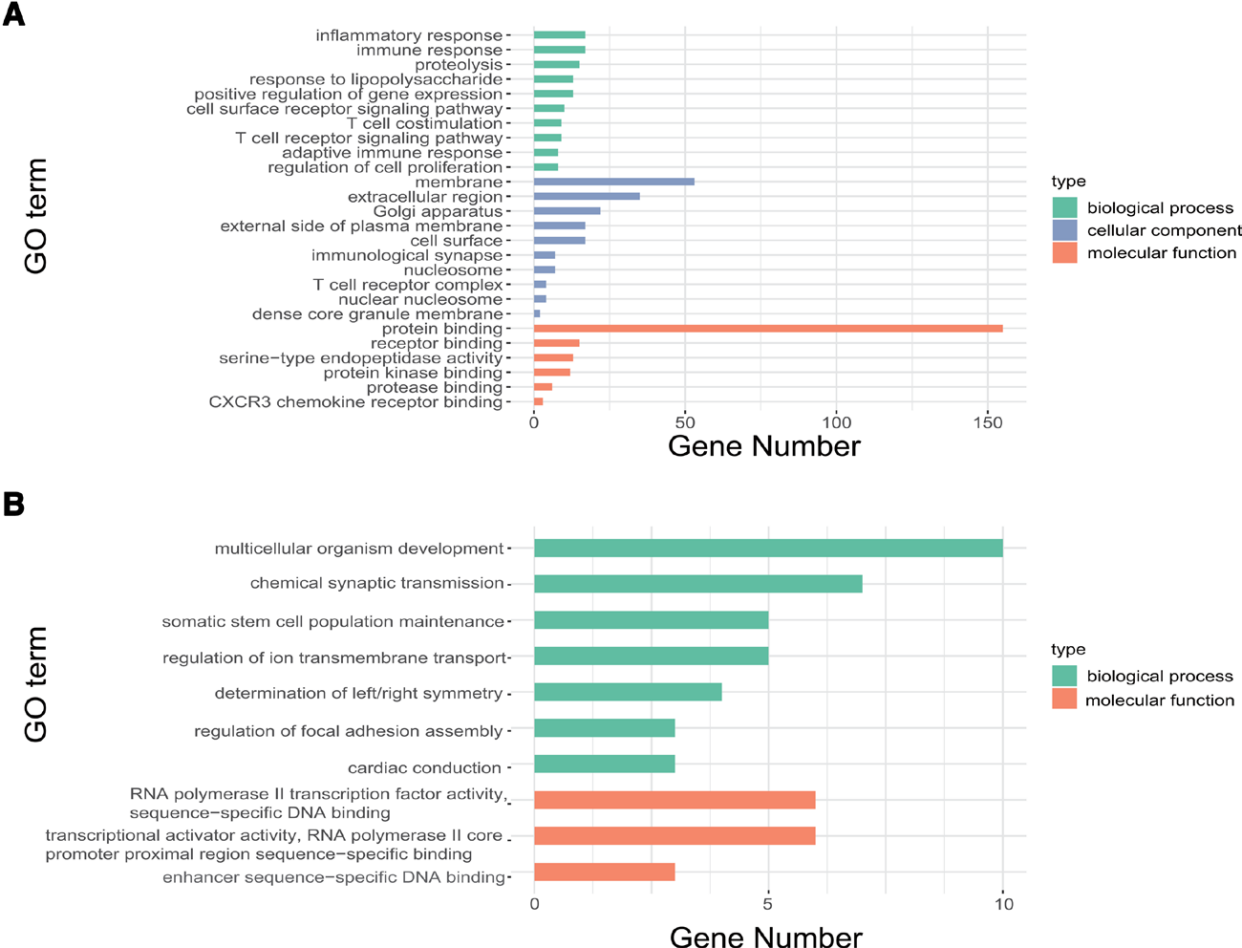
GO enrichment analysis of DEGs. (A) Enrichment of up-regulated DEGs. (B) Enrichment of down-regulated DEGs. DEGs = differentially expressed genes, GO = gene ontology.

To predict the functions and action pathways of DEGs, GO and KEGG analyses were performed on 342 upregulated DEGs and 202 downregulated DEGs using the DAVID database, respectively (*P* < .05 was considered meaningful, and only the top ten were shown when there were many results). The results of GO analysis showed that in BP, upregulated DEGs were associated with inflammatory response, immune response, proteolysis, response to lipopolysaccharide, positive regulation of gene expression, cell surface receptor signaling pathway, T cell costimulation, T cell receptor signaling pathway, adaptive immune response, regulation of cell proliferation, and other processes; in CC, upregulated DEGs constituted membrane, extracellular region, Golgi apparatus, external side of plasma membrane, cell surface, immunological synapse, nucleosome, T cell receptor complex, dense core granule membrane, protein complex involved in cell adhesion, etc; in MF, upregulated DEGs were in the process of protein binding, receptor binding, serine-type endopeptidase activity, protein kinase binding, protease binding, and CXCR3 chemokine receptor binding (Fig. 3A). Downregulated DEGs in BP are involved in processes such as multicellular organism development, chemical synaptic transmission, somatic stem cell population maintenance, regulation of ion transmembrane transport, determination of left/right symmetry, regulation of focal adhesion assembly, and cardiac conduction. The downregulated DEGs were also associated with RNA polymerase II transcription factor activity, sequence-specific DNA binding, transcriptional activator activity, RNA polymerase II core promoter proximal region sequence-specific binding, and enhancer sequence-specific DNA binding in MF (Fig. 3B).

**Figure 4.**
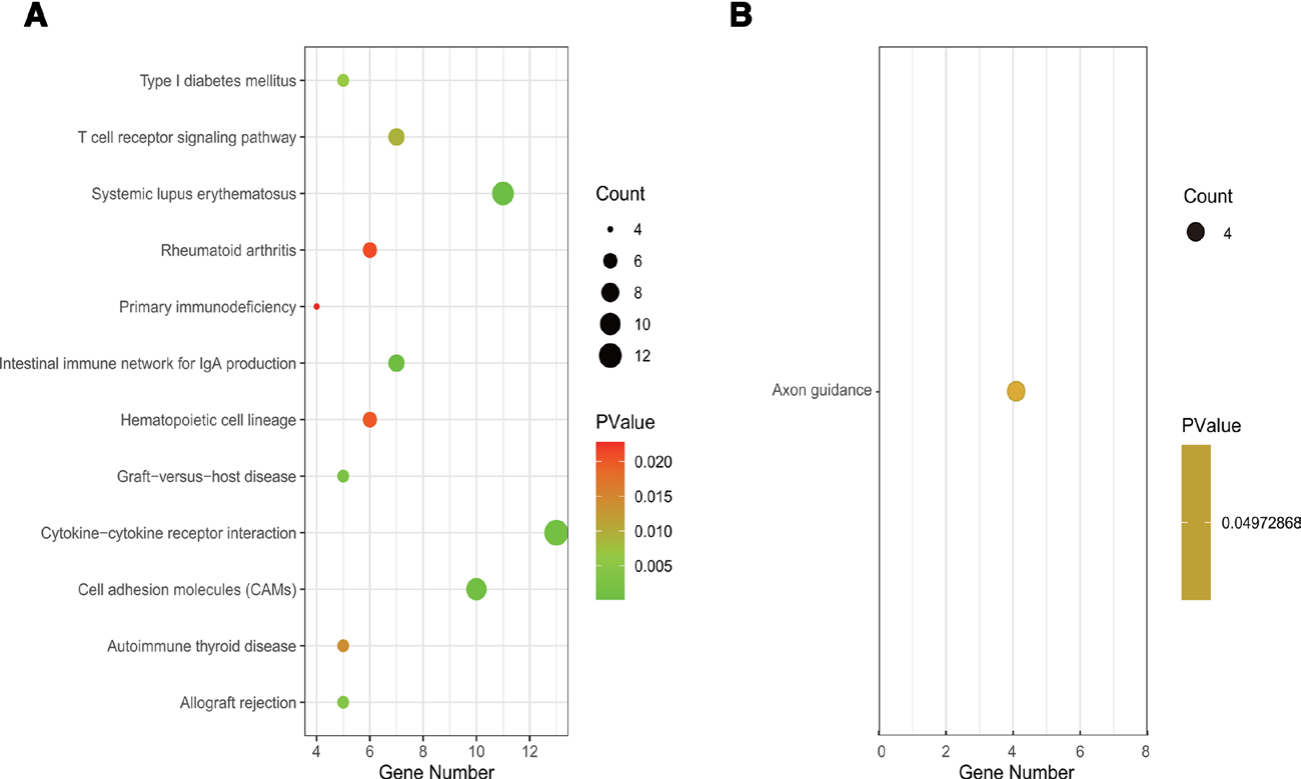
KEGG enrichment analysis of DEGs. (A) Enrichment of up-regulated DEGs. (B) Enrichment of down-regulated DEGs. DEGs = differentially expressed genes, KEGG = Kyoto encyclopedia of genes and genomes.

KEGG analysis was conducted in this study. The results showed that the related signaling pathways of upregulated DEGs were mainly concentrated in the cytokine-cytokine receptor interaction, systemic lupus erythematosus, cell adhesion molecules (CAMs), intestinal immune network for IgA production, T cell receptor signaling pathways, hematopoietic cell lineage, rheumatoid arthritis (RA), graft-versus-host disease, allograft rejection, type I diabetes mellitus, autoimmune thyroid disease, and primary immunodeficiency (Fig. 4A). Downregulated DEGs were enriched in only 1 pathway, axon guidance (Fig. 4B).

### 3.4. Construction of the PPI network and screening of hub genes

**Figure 5.**
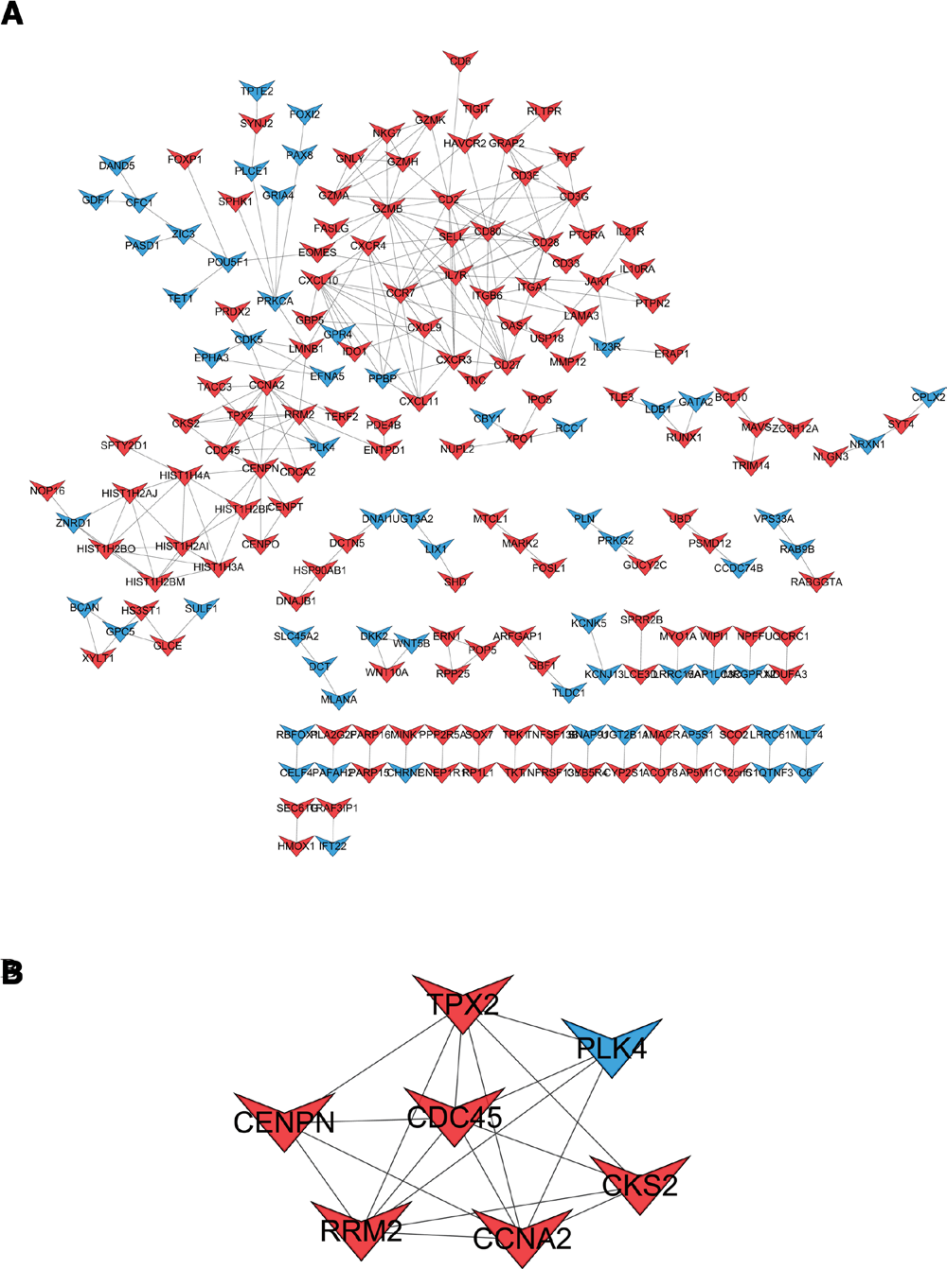
PPI network. (A) PPI network was constructed using all DGEs, with red for up-regulation and blue for down-regulation. (B) Application of MCODE plugin to screen out hub genes, the highest MCODE score = 6. DEGs = differentially expressed genes, PPI = protein-protein interaction.

The screened DEGs were uploaded to the STRING database to obtain PPI information, and the results were visualized using Cytoscape (Fig. 5A). The Mcode plugin was used for cluster analysis, and the module with the highest score was selected as the hub gene, namely, CENPN, CKS2, PLK4, RRM2, TPX2, CCNA2, and CDC45 (Fig. 5B).

### 3.5. Validation of GSE65127 dataset

**Figure 6.**
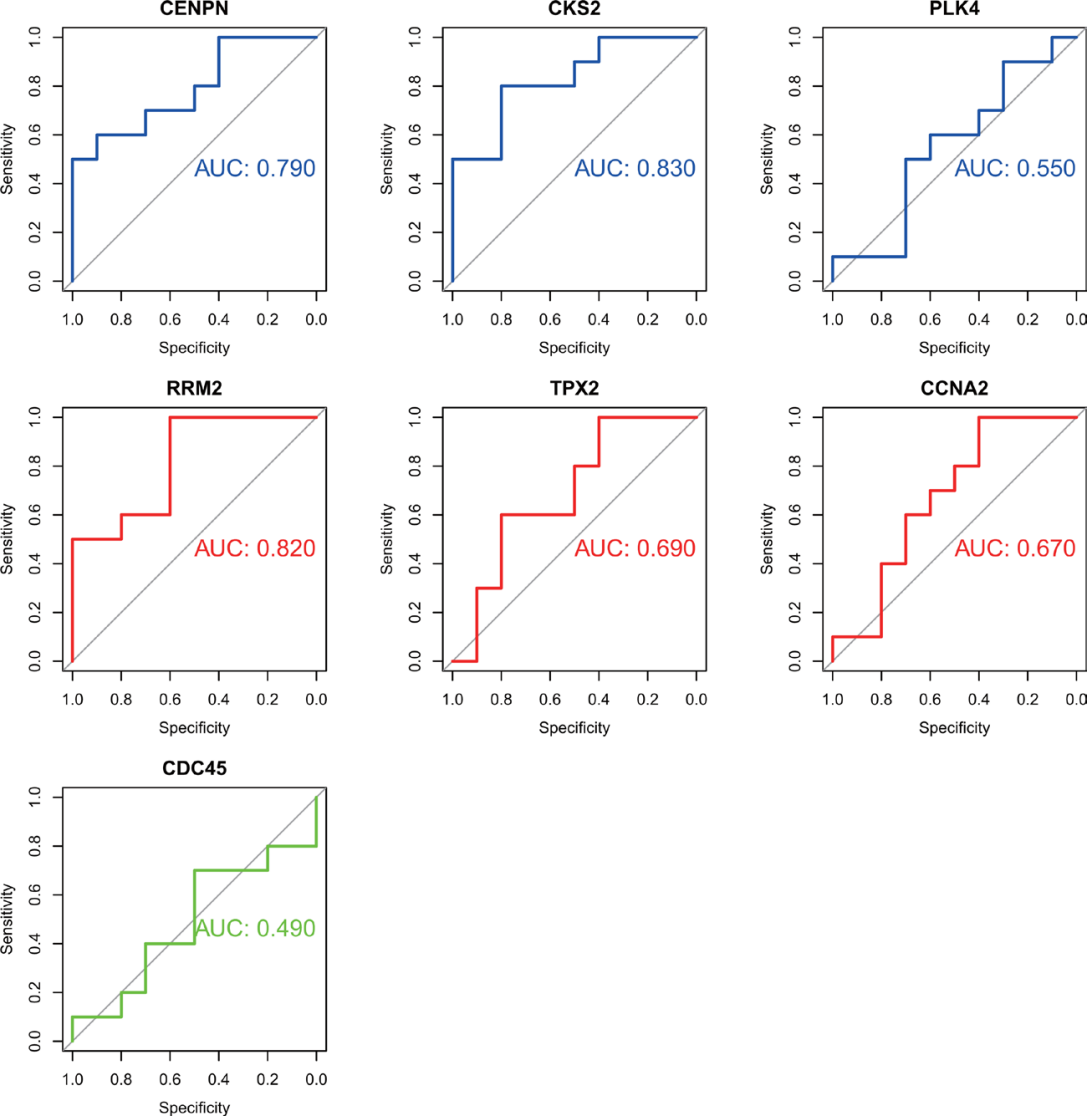
ROC of hub gene in GSE65127 dataset. The AUCs of CKS2 and RRM2 were > 0.8. AUC = area under the curve, ROC = receiver operating characteristic curve.

To analyze whether the screened hub genes were reliable, we used the GSE65127 dataset for verification, and 7 hub genes were selected using the pROC package in R (Fig. 6). Genes with an AUC > 0.8, namely CKS2 and RRM2, were selected for further analysis.

### 3.6. Immune cell infiltration analysis

**Figure 7.**
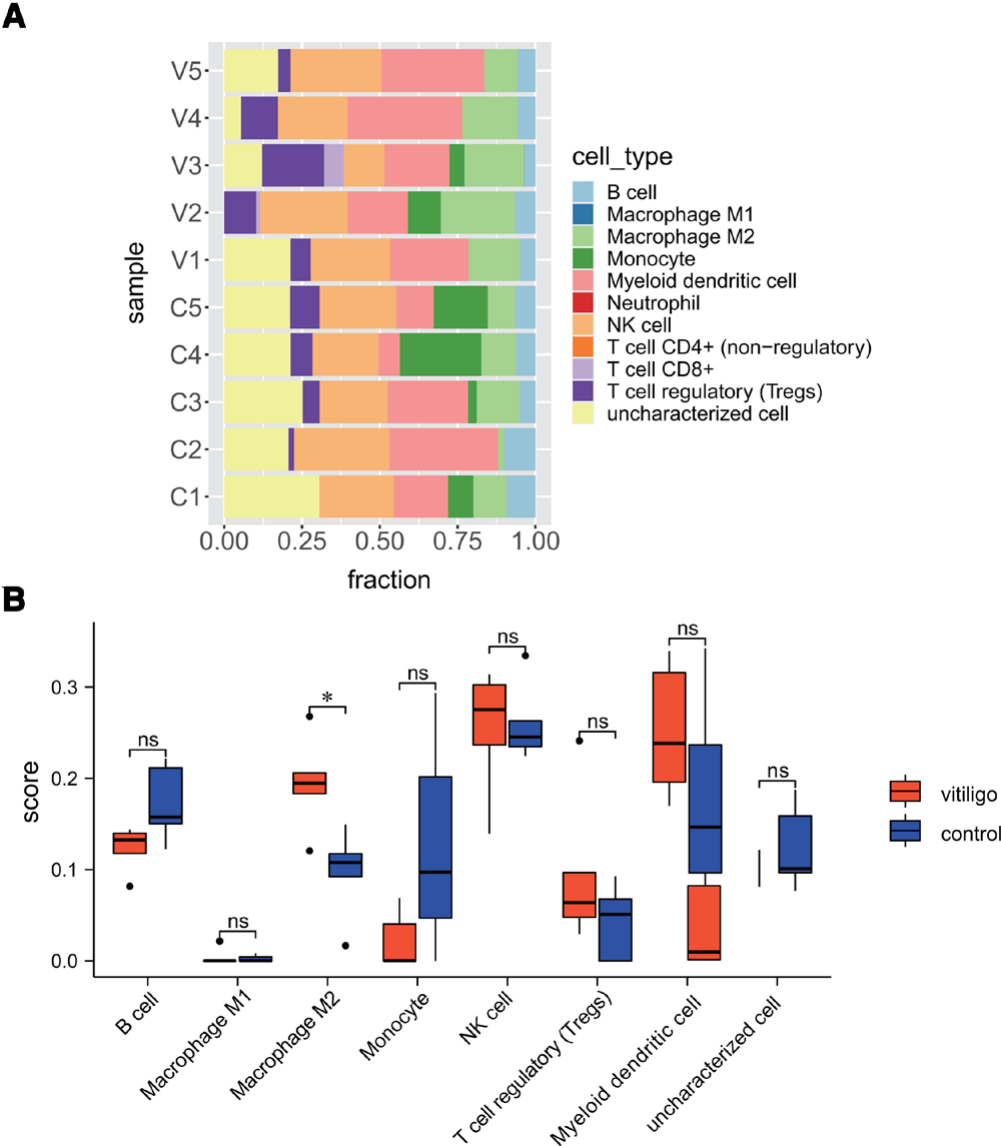
Immune cell infiltration. (A) Overall distribution of immune cells in 2 skin samples. (B) Analysis of the degree of immune cell infiltration in 2 skin samples.

We also analyzed differences in immune cell populations in skin lesions between patients with vitiligo and healthy controls. The cell populations in the 2 groups were evaluated using the quantiseq method, and the data were visualized. The bar chart clearly shows the distribution of the different cell types in the 2 groups of samples (Fig. 7A). Further statistical analysis of the degree of cell infiltration in the samples showed that macrophage M2 infiltration was obvious in the 2 groups (*P* < .05), and no significant differences were found among the B cells, monocytes, NK cells, Tregs, and dendritic cell groups (Fig. 7B).

### 3.7. Relationship between cell infiltration and CKS2, RRM2

**Figure 8.**
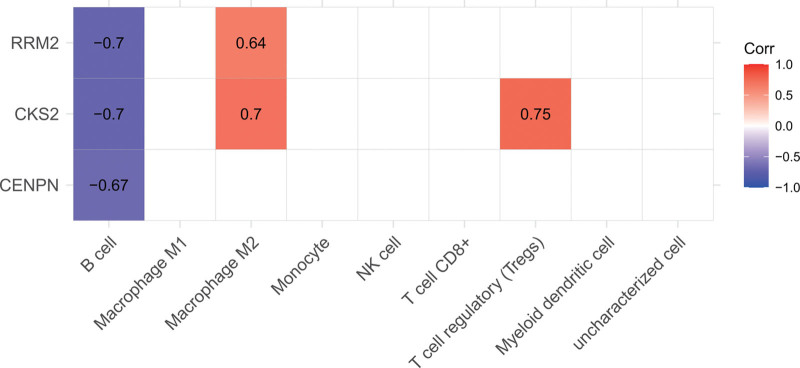
Correlation between CKS2, RRM2 and immune cells, respectively.

Through the above analysis, it was found that there were differences in the abundance of M2 macrophages between the 2 groups; therefore, we further explored the relationship between the cell population and the selected DEGs, namely CKS2 and RRM2. We plotted the relationship between genes and cell infiltration in the GSE53146 dataset and found that CKS2 and RRM2 were negatively correlated with B cell abundance and positively correlated with macrophage M2 abundance, and CKS2 was positively correlated with Treg cell abundance (Fig. 8).

## 4. Discussion

Vitiligo is characterized by patchy leukoplakia. It is a depigmented disease that can repigment, but is prone to recurrence.^[4]^ Although there are various treatment options for vitiligo, due to individual differences, high recurrence rates, and other factors, patients experience a heavy psychological burden and economic pressure.^[1]^ Therefore, it is necessary to identify new and accurate biomarkers of vitiligo. To achieve this goal, this study used bioinformatics to scientifically analyze the sequencing information of patients, screen out the core genes, identify markers that are meaningful for the occurrence and development of the disease, and provide a theoretical basis for understanding the pathogenesis and molecular targeted therapies.^[6]^

Firstly, the GSE53146 dataset was downloaded from the GEO database and 544 DEGs were obtained, of which 342 were upregulated and 202 were downregulated. Second, GO analysis was performed on all DEGs. The results showed that vitiligo was associated with immune and inflammatory reactions at the cellular level. Further KEGG results suggested that vitiligo is related to type I diabetes, autoimmune thyroid disease, RA, and other autoimmune diseases. According to the STRING database, a PPI network was constructed for 544 DEGs to find the connection between genes, and the MCODE plugin was used to screen out the important modules composed of 7 genes (CENPN, CKS2, PLK4, RRM2, TPX2, CCNA2, and CDC45). Finally, in the GSE65127 dataset, we applied ROC to verify the above 7 genes, identified genes with AUC > 0.8, and obtained CKS2 and RRM2, so that they can be used as key genes of vitiligo for further research.

The CDC28 protein kinase regulatory subunit 2 (CKS2) is a member of the human CKS family.^[9]^ CKS2 is highly conserved^[10]^ and can bind to the catalytic subunit of cyclin-dependent kinase (CDK), which is crucial for cell cycle regulation^[11]^ and is also closely related to embryonic development and somatic cell division.^[9]^ Previous studies have mostly focused on tumors and found that CKS2 is involved in biological processes such as DNA repair, the P53/P21 signaling pathway, and Akt phosphorylation.^[9,12,13]^ Ji et al found that in hepatoma cells, the expression of CKS2 was negatively correlated with the content of PTEN protein, and PTEN, an important molecule of the P53 signaling pathway, was related to the activity of P53 protein. The results showed that high CKS2 expression could inhibit the activity of P53 and participate in the progression of liver cancer.^[14]^ In patients with vitiligo, Ultraviolet B (UVB) radiation is a commonly accepted treatment for patients with vitiligo. Su et al demonstrated that UVB activated p53, which caused the downregulation of transient receptor potential cation channel subfamily M member 1 (TRPM1); thus, the expression of microRNA (miR) -211, encoded by the intron of TRPM1, was downregulated. MiR-211 changed the ability of melanocyte migration ability by targeting matrix metalloproteinase 9; therefore, CKS2 may be a therapeutic target to improve repigmentation in vitiligo patients.^[15]^ Previous studies have found that with the upregulation of CKS2, the mRNA of CDK2/4/6 proteins inhibited by the P21 protein gradually increased,^[14]^ and CDK2 was involved in the development of melanoma by regulating the G1/S and G2 phases of the cell cycle.^[16]^ Similarly, melanocyte loss due to the abnormal expression of CDK2 was observed in vitiligo patients, but the exact mechanism is unclear.^[17]^

Akt phosphorylation is closely associated with cell proliferation and apoptosis. Lin et al found that the protein expression of CKS2 was positively correlated with Akt phosphorylation.^[12]^ Akt can further promote IKK phosphorylation and activate the NF-κB pathway, which promotes the release of CXCL16 and IL-8 by keratinocytes, and an increase in CXCL16 can lead to the migration of CD8 ^+^ T cells in patients with vitiligo.^[13]^ We speculated that CKS2 might participate in the development of vitiligo through an immune response. Therefore, this study further analyzed the level of immune cell infiltration in the 2 groups, and the results showed that there was a significant difference between M2 macrophages. The correlation between screening genes and immune cells was evaluated, and CKS2 was found to be related to the abundance of B cells, M2 macrophages, and Tregs.

Ribonucleoside diphosphate reductase subunit M2 (RRM2) is a catalytic subunit of ribonucleotide reductase and a rate-limiting enzyme for DNA repair and synthesis. It plays an important role in cellular processes such as proliferation, migration, and angiogenesis.^[18,19]^ It has been reported that RRM2 is involved in the pathogenesis of various cancers, such as breast cancer, glioma, multiple myeloma, retinoblastoma, etc.^[19–22]^ Duxbury et al showed that overexpression of RRM2 activated the NF-κB pathway and increased the invasion of pancreatic cancer cells, while silencing the RRM2 gene could weaken NF-κB activity.^[23]^ In breast cancer, the NF-κB pathway is the main pathway affected by RRM2 overexpression, which could induce higher migration characteristics,^[24]^ while the NF-κB pathway is also involved in the pathogenesis of vitiligo. Chen et al found that NF-κB signaling in KCs promoted the expression of IL-15 and the transpresentation of IL-15, which in turn contributed to the activation of CD8^ + ^T cells and induced adaptive immunity in vitiligo.^[25]^ Zhuang et al verified that NF-κB stimulates the production of IFN-β, which leads to the secretion of CXCL10, and that excess CXCL10 in the epidermis plays a key role in the transport of autoreactive CD8^ + ^T cells, which further leads to the death of melanocytes in vitiligo.^[26]^ Therefore, we speculate that the upstream molecule, RRM2, of the NF-κB pathway plays an important role in the pathogenesis of vitiligo.

In non-neoplastic diseases, researchers have also actively explored RRM2. Studies have shown that fibroblast-like synoviocytes (FLS) promote the development of RA through the production of proteases. When RRM2 containing small interfering RNA was sent to RA-FLS, they found that the level of apoptosis increased and the production of cell proliferation and proinflammatory factors decreased, indicating that silencing RRM2 improved the therapeutic effect of RA.^[27]^ RA is an autoimmune disease that is closely related to vitiligo. Such individuals have a higher risk of developing vitiligo.^[5]^ Therefore, we speculated that RRM2, as an effective treatment target for RA, is also important for vitiligo. MiR-211 is involved in many cellular processes such as pigmentation. Another study found that the expression of RRM2 was upregulated in the skin lesions of vitiligo, and its 3’-UTR region had an miR-211 binding site,^[28]^ further verifying that *RRM2* could be used as an effective marker of vitiligo.

However, there are still some limitations to this study. First of all, sample size we used is small, and the results may have limitations, so we verified them using another dataset. Secondly, this study is based on bioinformatics and obtains related disease-causing genes, then we will carry out experimental verification at the cellular and animal levels.

## 5. Conclusions

This study briefly discussed the biological processes involved in CKS2 and RRM2. Some pathways and cellular activities overlap with the pathogenesis of vitiligo. Therefore, CKS2 and RRM2 could be used as potential markers for vitiligo.

## Author contributions

Feng Zhang conceived of and designed the experiments. Yu Miao, Dongqiang Su, Qian Fu, Taoyu Chen, and Yanqi Ji downloaded and analyzed the data. Yu Miao wrote the draft manuscript and all authors approved the manuscript for publication.

**Conceptualization:** Feng Zhang.

**Data curation:** Yu Miao, Dongqiang Su, Qian Fu, Taoyu Chen, Yanqi Ji.

**Formal analysis:** Yu Miao, Dongqiang Su, Qian Fu, Taoyu Chen, Yanqi Ji.

**Funding acquisition:** Feng Zhang.

**Validation:** Yu Miao, Dongqiang Su, Qian Fu.

**Visualization:** Yu Miao, Dongqiang Su, Qian Fu.

**Writing – original draft:** Yu Miao, Feng Zhang.
